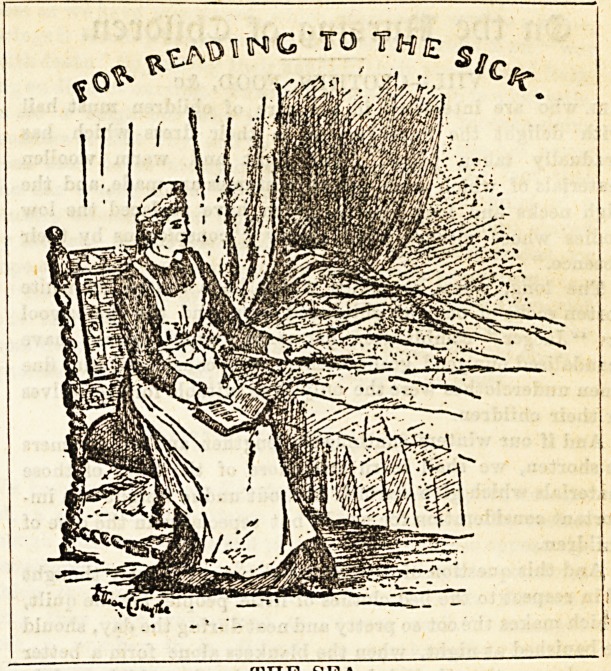# The Hospital Nursing Supplement

**Published:** 1892-04-16

**Authors:** 


"The Hospital" Nursing Mirror.

Being the Extra Nursing Supplement of "The Hospital" Newspaper.

Contributions for this Supplement should be addressed to the Editor, The Hospital, 140, Strand, London, W.C., and should have the word "Nursing" plainly written in left-hand top corner of the envelope.

### En Passant.

STOCKHOLM NURSES.—The nurses at the Sophiehemmet at Stockholm consist of sixty-four nurses and probationers. Fourteen of these are always available for cases outside the hospital. The staff is likely to be augmented shortly, for the Queen wishes the poor as well as the rich to be provided with nurses who can go to their homes.

TWENTY YEARS AGO.—We scarcely realise how quickly all sanitary ideas have changed for the good and young nurses who see the apple-pie order of hospital life, and the antiseptic method in full swing, little think the struggle it was for their pioneers to combat death and disease. Only twenty years ago in one of our largest lying-in institutions the women were all confined in feather beds!

HOW TO MAKE MONEY.—The Influenza "germ" has left an after trouble which seems more serious than the original affection. We hear of thirteen applications for "mental nurses," and twelve of the cases, requiring immediate skilled treatment, are directly traceable as due to recent attacks of influenza. Another developement connected with this "plague" is of a more humiliating character, for all true nurses will blush for its existence. We are told of a West-end private nursing institute where the salaries paid to each nurse is never over £24 per annum, and where, during the sudden pressure, the charges to the general public were raised from two guineas to four guineas per week. It seems incredible that such a proceeding could take place in charitable England, and that any head of an institution could be guilty of making capital out of a national scourge such as this, but "facts are stubborn things," and it was evidently quite time for nurses to co-operate for their own protection when such iniquitous private "farming" goes on.

SYMPATHY.—A gentleman went into one of the hospitals the other day to see after one of his workmen, who had met with an accident, and asked the nurse how he was getting on, and the nurse, in an off-hand manner, just answered his question with "he has had concussion of the brain, and has a cut here," at the same time carelessly letting her hand touch the place, which caused the man to shudder from the pain. The visitor then explained that he was the man's employer, and he wished to do or give anything which would help the man to recover. Whereupon the nurse, on hearing the story of the accident, became quite interested in her patient, and "I couldn't help thinking," said the visitor, "that a good many nurses look upon their patients as 'cases' only, and forget that they are human beings also. The nurse seemed to take no interest in the man till I explained matters." We do not believe there are many nurses of this pattern, and blunt manners often lead to wrong impressions; but it is well to remember that a nurse is used to constantly having new patients, and an accident brought in is quite an ordinary occurrence to her, while to be in a hospital ward for the first time and to awake perhaps from unconsciousness to strange surroundings is an unpleasant novelty to the patient. Nurses scarcely know the bare outlines of their patients' lives; they know nothing of their hopes and fears, their anxieties of how their homes and children are faring during their enforced absence. Kindly interest and sympathy are easy to give and they are as essential to a sick man's recovery as the most skilled nursing.

THE REGISTRATION OF NURSES.—The following note appears in the *British Medical Journal*, of the 9th inst., which we think it only just to reproduce: "Dr. Bedford Fenwick writes to assure us that it was never contemplated to forward copies of the petition in favour of the granting a charter to the Royal British Nurses' Association to the branches of the British Medical Association." We fully accept this assurance; the occurrence at the branch to which we referred must be taken, therefore, to be an isolated an unpremeditated instance.

"PUFFS."—A correspondent is not pleased because in one of our issues we pronounced a private asylum to be "the most comfortable asylum we have ever been over," and we thought the arrangements of the entire place good, and we said so. And by these remarks we are thought to have infringed some unwritten law, and to have given the asylum what is known to the advertiser as a "puff." Perhaps our correspondent has noticed that from time to time we give descriptions of various asylums, as well as of other institutions, noting how their buildings and sanitary arrangements are kept up to the times, marking retrograde movements, noting improvements of every sort, whether with regard to attendants or the treatment of the patients. Does our correspondent think these items and details are the overflow from an irresponsible and highly imaginative pen? They are, in point of fact, the result of personal observations of some one of our staff, wh goes to see, and not to hear, what is being done. This infringes no law; it does but give honour to whom honour is due, and blame where blame is due. If all just merit were hidden for fear that the mention of it would be considered a puff, improvement would vanish, and "dead level" would be the order of the day.

EAST-END MOTHERS' HOME.—The annual meeting of this institution was held at Lowther Lodge, Kensington Gore, and the Archdeacon of London and Canon Blackley, Dr. Clement Godson and Mrs. Stuart Wortley were some of the speakers who testified to a year's good work. It will be remembered that Mrs. Ashton Warner resigned her appointment there in October to go and practice her profession in New York. Miss Blomfield is now the Lady Superintendent. This is the only institution of the kind in East London, and Canon Blackley dwelt upon the help it needed in consequence, and also on the influence for good which the sojourn in the Home had on the lives and character of the poor women who are benefited. We, who live in the other end of the town, scarcely realise what it is to be at the most critical moment of our lives needing quiet above everything, living in the same room with the entire family, a woman probably working a sewing-machine above our head, and a drunken, quarrelling couple living in the room next door. This is not an exaggerated picture. An out-patient department has been started in connection with the Home, and women are attended by the midwives for the sum of 3s. 6d., which is much less than that charged by the ignorant so-called midwives, and this promises to be a great success, and affords a greater opportunity for training midwives. Two new pupil midwives are joining this week. Dr. Godson testified to the satisfactory working of the Home, and said that everything was well up to date, and that the "incubator" was working splendidly. One point that other institutions might well copy is that none of the patients' clothes are allowed in the Home. They are kept in a separate place altogether, and the danger of infection from clothes is no light one.

### Ventilation, Disinfection, and Diet.

By P. Caldwell Smith, M.D.

Ventilation: Composition of the Air—Ozone—Impurities —Bacteria—Gaseous Impurities—Carbonic Acid—Vitiation of the Air by Respiration.

Ventilation, disinfection, and diet and dietaries, may properly be said to belong to the region of preventive medicine, that is to the prevention rather than the curing of disease; but I hope to be able to show that a knowledge of these important subjects also enters largely into your duties as nurses in the treatment of disease.

Without pure air and proper food a patient cannot be expected to recover so rapidly, while with these, he is, as far as hygiene is concerned, in the most favourable condition for the employment of other means, therapeutic or surgical, for his recovery.

First, then, in regard to ventilation; but it is necessary, before entering on the question of ventilation of rooms, &c., to know the composition of the air we breathe, and what produces the necessity for ventilation at all.

Air, and I mean by this ordinary pure air taken from a free open space, consists of a mixture of the following: Oxygen, 20·96 per cent.; nitrogen, 79 per cent.; carbonic acid ·04 per cent., or ·4 per 1,000, which is perhaps more easily remembered, with a variable proportion of ammonia, organic matter, ozone, and suspended matters, all of which, with the exception of ozone, may be reckoned as impurities. The effect on the health will depend on the amount of these impurities plus the carbonic acid. This latter, carbonic acid, may be called the most important impurity, not of itself, but because it is by the estimation of the amount of this that we can most easily gauge the amount of organic matter present.

Oxygen is the essential component of the air, while nitrogen is simply the vehicle for the oxygen. The oxygen does not vary very much, the percentage amount being generally at a maximum when it reaches ·32—that is to say, a room is hardly fit to live in when the amount of oxygen reaches 20·64 per cent.

Nitrogen, which is a colourless and odourless gas, is quite incapable of sustaining life, and if an animal be placed in a jar of this, it dies almost at once from suffocation. As I have said, the amount of carbonic acid$_2$ in average pure air is ·04 per cent., or ·4 per 1,000. Ozone is really condensed oxygen, or oxygen in a very active condition. It is more abundant in mountain air than in that in the valley, and it is found in considerable amount in the air at the seashore. It is seldom found in the air of inhabited rooms, as it is very quickly destroyed by the impurities in the air. It is to be remembered that ozone, although popularly talked of as being health-giving, &c., is in reality a very deadly poison, as oxygen, when it contains $\frac{1}{240}$ part of its volume of this substance is fatal to all animals.

Let us now consider the impurities of the air, and first as to suspended matters. The larger particles are composed of dust or sand taken up from the ground by the wind and carried by it for long distances. The smaller particles are mainly animal and vegetable organisms, and it is almost impossible unless at very high altitudes as on the tops of mountains to get air free from these impurities. The larger forms of animal organisms found retained their vitality even when dried for a very long time, while portions of animals, as flies and spiders, are also very frequently found. From the vegetable kingdom we find in air seeds and vegetable fibres, while in spring and summer pollen grains are carried by the wind long distances. Of course, in towns and cities, particles of tar and soot are very common, these being formed from the combustion of coal.

Lately much attention has been paid to bacteria or micro-organisms in air by various authorities in this country and in Germany. These are of the very greatest importance, as they are the agents which, in many instances produce zymotic or infectious diseases. All the organisms which are found in the air do not produce disease, or no one could possibly escape. It is only a very few which are what are called pathogenic or disease-producing. One observer found that the number of organisms per cubic meter was as follows:—

| | |
|---|---|
| Sea air | 6 |
| Air of high mountains | 1 |
| ,, saloons of ships | 60 |
| ,, new houses in Paris | 4,500 |
| ,, old Paris houses | 36,000 |
| ,, Hopital de la Pieté | 79,000 |

Even where they reach the enormous amount stated in last example, there might possibly be very few disease-producing germs, although it is to be remembered that the probability is that the greater the number of germs the more impure will the air be.

Some of these disease-producing bacteria can be carried by the air to a certain distance. In the case of cholera the germs may be carried in particles of dry excreta, while in cases of scarlet fever and small-pox the dried epidermis is the carrying agent.

In houses the air will, besides containing the substances already mentioned, also contain particles of wool and cotton fibres, starch grains and scaly epithelium, or the dried scurf skin from hands, face, and head.

In sick rooms, hospitals, and rooms occupied by persons suffering from skin diseases, other substances will be found, as the spores of ringworm, and the bacillus of tubercular disease. This latter is invariably present, as has been proved by frequent experiments, in all rooms inhabited by persons suffering from tubercular disease of the lungs, while houses in which no cases of this disease exist are generally free from its presence. This is a most important thing to remember, as you will see when I come to speak of disinfection in this most fatal disease.

Besides these suspended matters, air also contains frequently some gaseous impurities. Carbonic acid gas although present in the proportion of ·4 per 1,000 parts in average pure air becomes an abnormal constituent or impurity when it reaches more than ·5 per 1,000 parts. I shall speak of this substance more in detail when dealing with air vitiated by respiration. Other gaseous impurities are carbonic oxide, sulphurous acid, hydrochloric acid gas from alkali works, a gas which is frequently found, though in very small quantities, in the air of some parts of Glasgow; ammonia, nitrous and nitric acids. There are also present, in varying quantities, organic vapours derived from the decomposition of animal matters.

I have now to answer the question, What produces the necessity for ventilation? It is that the air we breathe is constantly being polluted by respiration and combustion, and must be renewed from time to time, and kept as nearly as possible in an average condition of purity.

Let us first consider the vitiation of air by respiration. An adult man, at rest, gives off on an average ·6 of a cubic foot of carbonic acid per hour, with a certain proportion of moisture or watery vapour. From the skin and lungs of an adult 25 to 40 ounces of water pass off in 24 hours. The amount of this depends largely on the temperature of the air, a very much larger quantity being thrown off in hot weather. There is also given off a certain amount of organic matter, this being distinctly perceived by the sense of smell when the carbonic acid reaches ·7 per 1,000, and when it is 1 per 1,000 volumes the odour is very strong. I am sure some of you have had occasion to enter a close, unventilated, and overcrowded apartment in the morning, and have felt this organic smell. It is in an atmosphere impregnated with this that typhus fever revels, and it is probable that it is only there that the germ of this disease can breed. This organic matter may be also found on the walls, and as a consequence these walls, if not thoroughly cleansed, become a source of danger to the occupants of the room.

### Asylum Attendants.

Our readers will remember some weeks back we received two or three letters about the "Morison Prizes" which were awarded this year to Robert Elmslie, head attendant at Montrose Royal Asylum, for long and faithful service, and to Helen Matthew, head nurse at Saughton Hall, for zealous and faithful service, and specially "for having gone through a systematic course of training as a nurse in a general hospital and having gained a certificate." Every year brings a little advance in the attention paid to the necessity of training asylum attendants, and gradually Medical Superintendents and Committees will begin to see that knowledge of the care of ordinary ailments is as necessary in attendants on the insane as it is for those on the sane; it is evident that Dr. Batty Tuke, who, as Morison lecturer this year, recommended who the recipient of the prizes should be, fully appreciated this. A nurse, seeing notice of the prizes in The Hospital, wrote to us saying what a tendency such things have to increase the interest in nursing the insane, and that numbers of attendants are ambitious to gain such prizes, giving another proof that training is wished for by attendants, a fact which reaches us from all sides, and that they feel the necessity of it themselves brings us already some way on the road of progress. The exact particulars of "The Sir Alexander Morison Prizes" have been sent us by a correspondent. They are confined to male and female attendants in Scotland, and they are in the hands of the Royal College of Physicians at Edinburgh. They are given for meritorious attendance on the insane. Two prizes are given yearly, each consisting of £3 in money, a certificate of merit, and a silver medal and ribbon. All applications for the prizes must be sent to P. A. Young, Esq., Royal College of Physicians, Edinburgh, before the close of the year; and so English nurses are debarred from this competition. Berrywood Asylum, Northampton, is one of the lucky asylums where lectures and instruction are given to the nurses, and we gladly record that all the nurses who obtained certificates last year for "first aid," have this year passed the examination for "nursing" successfully, and in addition, ten more nurses passed "first aid" examination, thanks to the energy of their medical officer. Who does not know the pleasure of possessing a testimony in black and white, given by some competent person, to one's efficiency in some one thing; it is not enough to feel one is proficient, it is the possession of a document to that effect, which gives confidence, not placid lazy contentment, but a longing to go further and gain more. These ambulance examinations are within the reach of everybody, of every race, provided an instructor can be found to give up his time.

The number of competitors for the Medico-Psychological Association certificates was proof that a need was felt for something of the kind, and while just a few here and there stir up the struggle after fuller tuition and knowledge, the numbers gradually swell.

This subject reminds us that perhaps some of our readers may never have heard of a fund called the Elliot Charity, which is for the benefit of persons employed in the care and control of any insane person in any licensed house in England only, and who, whilst so employed, has become incapacitated by sickness, accident, old age, or other infirmity from continuing in such employment. The forms of application must be applied for to the officers of the Commissioners in Lunacy, 19, Whitehall Place, S.W.

---

"In herself she dwelleth not,
No simplest duty is forgot;
Life hath no dim or lowly spot
That doth not in her Sunshine share.
She doeth little Kindnesses, which
Most leave undone or despair,
For naught that sets one heart at ease,
Is low esteemêd in her eyes."—*J. R. Lowell.*

### For Reading to the Sick.

### THE SEA.

How varied are the aspects presented by the ocean! One day it delights us as it ripples and dances in the sunshine, with a thousand dimples smiling on its waves, on the next we tremble, for it is raging like a furious madman, destroying all before it. Yet in every mood it has a beauty and fascination for most of us, though the sad moaning of its pitiless beat on the shore may only recall lost youth, lost hopes, lost pleasures. How like is human life to the ocean!

In the Bible the sea is used, generally, as a type or figure of wickedness, and it is mentioned in the Revelation of St. John as one of the joys of the heavenly Jerusalem that there will be no more sea. The pious widow of a Cornish fisherman once said it was her greatest comfort to think of this, for the cruel monstor had swallowed up everything near and dear to her; father, husband, sons—all had gone down into its insatiable jaws, never to return till the sea gives up her dead.

We who are sailing prosperously on life's voyage, with everything fair around us, are apt to say in our hearts, "I shall never be removed, Thou God of Thy mercy hast made my hill so strong," while at the very moment, behold there ariseth a little cloud like a man's hand; quietly it comes, almost unnoticed, but presently the Heavens are black with clouds and wind and there is a great rain. It may be a little business trouble which startles our security and reminds us that we are not to live only for ourselves, or a slight illness makes us remember that life is but a preparation for an endless future. We dare not slight these hints lest a worst thing happen unto us. A dimmed sight, a worried brain, sickness of any sort, suffering, and sorrow are the clouds which hang over our lives and threaten that our sun shall go down ere it is day; they are the winds which blow and the rough waves which beat upon our barque. Shall we survive these storms, shall we safely reach the haven where we would be? Oh! happy we, if Christ be with us in the ship, even though He appears to be asleep and unconscious of our danger.

He is only waiting for us to rouse Him. He wants us to cry to Him with our prayers and our tears, "Help, Lord, or we perish!" When He sees we are in earnest, He will calm the storm with "Peace, be still." He will say to us, "My children, why are ye fearful? O ye of little faith. These storms are not sent for your punishment, but only to bring you to My feet. I have lavished My love upon you, but ye are cold and careless; do not murmur or repine at your sufferings, they are the marks of My hand to prepare you for life or for death. Whatever your Heavenly Father has in store for you, I will help you to bear it. With Me for your pilot your little vessel shall so pass through the waves of this troublesome world that it shall finally ride safely in the Harbour of Rest."

"Oh! when our life is clouded o'er,
And storm winds drift us from the shore,
Say, lest we sink to rise no more,
Peace, be still."

### On the Nursing of Children.

### VIII.—CLOTHES, FOOD, &c.

All who are interested in the care of children must hail with delight the improvement in their dress which has gradually taken place. The light and warm woollen materials of which such pretty costumes are made, and the high necks and long sleeves which have replaced the low bodies whose sleeves were chiefly "conspicuous by their absence."

The long, warm stockings which have ousted the white cotton socks of our own childhood days, and the lambswool or "Jaeger" combination garments, which would have scandalised our good grandmothers, who considered that fine linen underclothes were the only ones suitable for themselves or their children.

And if our winters continue to lengthen and our summers to shorten, we shall learn still more of the value of those materials which give warmth without undue weight—an important consideration for us all, but especially in the case of children.

And this question of weight is not yet sufficiently thought of in respect to the bed clothes of little people, for the quilt, which makes the cot so pretty and neat during the day, should be banished at night, when the blankets alone form a better covering, as they lie lightly over the sleeping child, and do not weigh him down as most counterpanes will.

All children should sleep in flannel nightgowns during the winter, and these can be made as pretty, if not even prettier, than calico ones. If subject to cold feet, knitted sleeping socks should be worn, but the latter must fit loosely, or else they may do more harm than good by their interference with the circulation.

If it is very cold weather, or if the child be delicate or chilly, a small blanket placed next to it is admissible and comfortable; but any heavy coverings are undesirable, and tend to make him restless and uneasy—this is proved if the removal of one blanket enables him to settle down into quiet and refreshing sleep.

Children suffering from rickets and from some other diseases habitually free themselves from their bedclothes; hence, to them, the value of flannel gowns and sleeping socks is very great, and these render the practice of sleeping outside the bed comparatively harmless as regards a risk of cold catching.

We have heard of a fanciful and, of course, wealthy lady who engaged a trained nurse merely to sit up at night and replace the clothes when her children kicked them off. As the children were healthy, we may rest confident that either their suppers or their bed coverings were of an injudicious nature, and that an alteration in these would have obviated the necessity for a night watcher.

It is a great mistake to let little children, or big ones either, go to bed with cold feet, for the discomfort keeps them wakeful and fidgetty, and this certainly does not improve either general health or temper. It is one of the points not sufficiently considered—one of the small miseries which need not exist—and yet it is one that is frequently overlooked in luxurious nurseries.

The ward nurse who makes children her especial study soon learns the care that is needed in watching over little feet confined to splints, and she packs the tiny toes into a nest of heated cotton wool if the hot-water tin does not suffice for their comfort.

We need hardly mention here, perhaps, the invariable rule that no tin or earthenware foot-warmer must ever find its way into a bed until it has been securely enveloped in a closely-fitting flannel case, and it is sometimes well, in addition, to put a fold of blanket between it and the child's sensitive skin.

From clothing and warmth we pass naturally to the subject of food, which has so much to do in the production of heat. Good and proper nourishment being an essential part of treatment, and coming into the province of nursing, of which Dr. Eustace Smith, in his introduction to his book on "Diseases of Children," says, "the details of nursing should always take precedence of those of drug giving."

In district nursing amongst the sick poor, the questions of warmth and food are perhaps the most difficult with which we have to deal, and it is only when we are able to administer, or to see administered, the proper amount of properly prepared nourishment, that we can feel any confidence that the child has some chance of recovery. Who can realise, as do the doctor and nurse, the value of hospital routine? the regular food given at stated hours and in proper quantities, for too much is as dangerous to a sick child as too little. How this contrasts with the haphazard feeding of the thoughtless, who give large quantities at long intervals, or else keep the unfortunate patient supplied constantly with things to eat, which, whether suitable or unsuitable in kind, are alike disastrous in quantity, giving no chance of rest to the already disorganised digestion.

In getting the proper nourishment not only swallowed, but enjoyed, the true nurse shines, for all her intelligence and tact are brought to bear upon the subject, and few indeed are the little ones who do not yield to her pleasant coaxing of their wayward tastes.

In the capriciousness of their appetites when ailing, children are all pretty much alike, but whereas an unusual dainty is easily suggested for the child of poverty, it is far more difficult to provide a novelty for the delicately nurtured child—but it can be done by the exercise of some ingenuity, and, in fact, we go further, and say that it must be accomplished; probably some quite simple article may answer the desired end.

We knew a dear little Irish boy who had won all hearts with his big, pathetic eyes and quaint little ways and words, and who, after being several times brought back from the gates of death by the surgeon's skill, was at last pronounced to be beyond any further human help, and though free from actual pain, grew daily weaker and daily more indifferent to his usual diet. He would occasionally take a little milk—out of native politeness "to please you," he said to his nurse—and she managed to give him a gratification in her turn, for one day a bright inspiration caused her to say, "Would Peter like a potato, a big one, all to himself?" He nodded acquiescence, being, like most children when very ill, sparing of speech, and he was soon provided with a fine mealy potato, in its skin and a plate, fork, butter and salt, with the result that it was not only eaten, slowly and enjoyably, but also the small patient was diverted and employed for nearly half an hour. This may read as a trifling indulgence of a dying child's fancy, but the humouring of the whim was as good for the nurse as it was for her little patient. It is but one of many instances which recur to the memory of those who have learnt to comprehend children's ways, and to feel how easy it is to give small pleasures.

To some of us may be given an instinct so reliable that we know how to satisfy, and therefore how to manage children, and this with but little effort to ourselves. To others it is as a difficult lesson, and one needing much study and many repetitions before it is mastered at last, for learnt it can be by all who are willing to try. Only they must start with respect and reverence, as well as love, for "the little ones"; they must not expect miniature men and women, but they will find something a great deal better. They will discover fresh, pure natures ready to be moulded into good and noble characters, a capacity for generosity and patience, hopefulness beyond description, and faith and affection without limit, for all who have charge of them.

When we find children untruthful, mean, and disobedient, before blaming them we should just think over their previous history, and we would find almost invariably that their circumstances, parents, or guardians are chiefly responsible for the development of the faults. If children are surrounded by pure and wholesome influences—and these include plenty of fresh air and rational play—they will develop in a correspondingly healthy fashion. If they are trusted they will probably repay the confidence placed in them, and they must not be tempted to the committal of follies by over-strictness or by suspicions. Their "being good" should be oftener taken for granted, and in nine cases out of ten they would reward the trust placed in them by proving faithful.

It is fear—the knowledge that they are liable to be blamed for trifles by those governors of their universe, "the grown-ups," which hurries short-sighted youngsters into deceits which shelter them for the moment, but immediately lower their moral state, of which deterioration the poor mites know nothing.

Therefore, let us aspire to rule the "little ones," whether in sickness or in health, with a kindly, gentle, wise-like sway—albeit a firm one—for, strangely enough, these small creatures have a scorn for weakness, which they are swift to detect in other people. They love the strength which compels obedience whilst it is merciful to their frailties, and which is always more ready to forgive than to punish.

### The Old and the New.

Dr. L. M. Griffiths, in the *Bristol Medico-Chirurgical Journal* writes: "If lives of mothers are to be saved by rigid attention to these details (viz., extreme cleanliness and rigid antiseptic method), it is obviously necessary that the women in this country who attend most of the midwifery should be of a higher order of intelligence than those with whom we are now practically acquainted, and something must be done in the way of legislation to prevent a woman entering on the responsible duty of midwifery practice with no other qualification than that of poverty or incapacity for earning a livelihood in any other walk of life." Only last week we knew of a case in a country village where the old midwife, a poor feeble old body, attended a woman in her confinement, and during the next few days attended two more cases; the first woman is dead of puerperal fever, another is recovering from it, and the other escaped. The midwife did all she could in that she sent for the doctor thinking the poor soul very bad, and the coroner put an end to her spreading infection more. But just to think of the terrible danger that may result from helpless ignorance! "They ought to do something to stop these old women nursing, as don't know much about it," said one of the villagers as we came from enquiring how the women fared, and so say we.

To quote further from Dr. Griffiths, "It is of the greatest importance to see that nurses and other attendants are very careful about all details, and that by their carelessness the doctor's work is not undone. Ahlfied considers that on these points the public should be instructed by oral and written communications." But who is to instruct the *poor* public? It is only as trained district nursing spreads that ignorance as regards infection can lessen, and thoroughly competent midwives will be the ones to lighten the darkness, for hundreds of working women prefer, and in all probability will continue to prefer, the services of their own sex to sending for "the doctor" in their time of need. It is gradually dawning on the public that something must be done to prevent ignorant women from performing duties which only the most skilled ought to attempt, but it is dawning none too soon. Such a case as we have mentioned, which practically wrecks a home, brings it forcibly before us, and the well-off public must "war with death" by putting their hands in their pockets and helping on district nursing. Practical demonstration is what tells, to see a neighbour who is usually slow to recover doing wonders in that way, and to hear her say that she has never been seen to so nicely before, coupled with the fact that she has a new nurse, will work wonders on the mind of a country woman. Until we can get ignorant midwifery stayed by the arm of the law—and this will be consummated we hope ere long—we must hope by comparison to conquer.

"Be not the first by whom the new is tried" says Pope, and it is this wariness that makes many a woman stick to the local Gamp—but the new, once given a trial, our old enemy, ignornace and disaster, will vanish for lack of trade.

### Hints to Nurses.

We have received from Egerton Burnett, Wellington, Somerset, a selection of patterns of their summer stuffs, varied enough both in pattern and price to suit the most oppositely constituted tastes and pockets and all possessing that attribute which the cautious purchaser dwells on with pleasure, viz., they are guaranteed to "wear well." Amongst such a number it is difficult to particularise, but we will begin with business first in the shape of an excellent blue serge called "The Dora," wide in width and moderate in price, which would make excellent dresses for those in our hospitals who still adhere to stuffs for Sisters or nurses' uniforms; the "Richmond" and "Royal" serges, a few pence dearer, are still better; the qualities run up to almost any price and are beautifully soft. A soft drab material called "The Knockabout," is strongly suggestive of holidays, and looks as though it justified its designation. The "Dorcas" and extra-strong dark blue serges ought to be remembered when winter comes again, and we are busy stitching for those who possess no warm clothing; they are so strong and so very moderate in price, and would be admirable, let us say, for the "Hospital" Christmas parcels! Small fry would be able to be cool and comfortable in frocks of "Royal" serge of quite pale colourings, or in "Empress" cachemir, which last would delight the heart of anybody clever at "smocking." Endless varieties of "summery" yet serviceable stuffs are among the patterns, but, as we said before, we cannot tell our readers about them all. And now for the practical purposes of those whose garments can vary little in shape or fashion; life is made up of little things, and if we have to lie for days and weeks in the same position, we do not despise the little variety afforded by a change of colour; the flannels and flannelettes are so woolly, and of all imaginable colours, that it would be easy to ring all sorts of changes on dressing-gowns, bed jackets, or nightingales.

### Marriage.

We learn by the last mail from Australia that Miss M. E. Stephenson, who was appointed Lady Superintendent of the Victorian Eye and Ear Hospital at Melbourne in November, 1890, has recently married the secretary of that Institution, Mr. T. G. Leslie.

### Appointments.

[It is requested that successful candidates will send a copy of their applications and testimonials, with date of election, to The Editor, The Lodge, Porchester Square, W.]

Charing Cross Hospital.—Miss J. Watson, who was trained at Leeds General Infirmary, has been recently appointed night Sister.

### Everybody's Opinion.

*[Correspondence on all subjects is invited, but we cannot in any way be responsible for the opinions expressed by our correspondents. No communications can be entertained if the name and address of the correspondent is not given, or unless one side of the paper only be written on.]*

### TALKING SHOP.

"Anti-shop" writes: Is there not a danger lest we, as hospital nurses, should allow our sympathies and interests to become narrowed by constant revolution around our local centre? Especially at our meal times I have noticed how alarmingly prevalent is the pernicious custom of "talking shop." Although our profession is an absorbing one, yet surely to eat of it, drink of it, by discussing the cases and their treatment is not conducive to the intellectual benefit of the community as a whole. All of us are liable at times to fall into this snare, forgetting that for such matters we may well find a more convenient season. That kind of light and cheerful conversation which S. Frances de Sales tells us the Greeks call *Eutrapelia*, is too much neglected amongst us. Surely it is not unworthy of effort to cultivate an interchange of opinion on matters of general interest, and to strive so to be enlarged. I should be glad to know whether the nurses of other large institutions have observed this tendency to narrow-mindedness, and if so, perhaps they would be willing to offer suggestions for the formation of a corrective (anti-shop) association.

### MRS. CREIGHTON HALE'S NEW BOOK ON MASSAGE.

Nurse Caller, Troy, U.S.A., writes: As a reader of The Hospital, it is clear to me that you are a true friend of nurses, and like to advise them for the best. I see Mrs. Creighton Hale is teaching two blind girls. I know she will succeed, for I am happy to say I am one of her pupils. I went all the way to London expressly to be trained by her, and from the splendid instruction I received I consider it the best money I ever spent. Hoping every nurse who wants to be thoroughly taught the art of massage will go where I did. I look always for Mrs. C. Hale's book on massage, which she was engaged on when I was in England. Is it yet published?

[*** The book will be published shortly after Easter, and may be obtained at 140, Strand, W.C.]

### THE NURSES' CO-OPERATION.

Miss James, of Philadelphia, sends us the following testimony to the invaluable services rendered by Miss H. Morten from the first in organising the Nurses' Co-operation, which we can most cordially endorse from our own knowledge of the fact: "I have before me the report of the Nurses' Co-operation, and while rejoicing in its success, notice that Miss Honor Morten's name does not appear among those who are credited with originating the scheme. To her I think much is due. Shortly after my arrival in this city from London in the year 1888, I wrote to Miss H. Morten about the Philadelphia Directory for Nurses. She at once replied, asking for further information about its working. Through the kindness of Dr. Weir Mitchell, President of the College of Physicians, and one of the original promoters of 'The Directory,' I was enabled to obtain all the necessary papers and forms; these, with all information, and their annual reports, I sent to Miss Morten. She frequently wrote me about her plans for starting a similar scheme, and during my visit to London in 1890, she sought at our interview to elicit every particular that would assist in starting the work. Miss Morten was untiring in her endeavours, and I was pleased to find some one more enthusiastic (if possible) than myself in their anxiety to see this 'new departure' established in 'Conservative' London. Miss Morten was, if I mistake not, hon. secretary. With best wishes for the continued success of the 'Nurses' Co-operation.'"

### HOLIDAY EXPERIENCES.

H.T. writes: In reference to an article headed as above, may I state that I met one of the nursing profession, who said, "I have never had a holiday I have enjoyed," while I replied, "I have never had one I cannot look back on with pleasure." I am convinced the chief thing nurses need is not the rest of body they long for so much on the eve of the holiday, but a complete change mentally. Rest is needed for the first two or three days, but after that the energetic mind and body, accustomed to exercise, needs variety and movement. If a nurse has no friends who look for her company, I can suggest two modes of spending a fortnight at the cost of £5. Let her get a companion of similar tastes, choose a rural country village, and after the rest take walks in every direction. Let her take a cheap ticket via Harwich and Antwerp, and see as much of Belgium as means permit. The names of cheap boarding houses would be received in reply to a query in The Hospital. One of Percy Lindsay's penny guides would suffice for other information.

### Greetings from Vancouver.

Sister Frances sends us a letter from Vancouver, telling of plenty of hard work, thanks to our old friend the influenza. She writes: "We are all nearly killed, and so glad to have a day or two of perfect rest. The two nurses are stealing time to sew a little every day, and so save the money we should have to pay if a woman were engaged, and this money will be our only means of giving an Easter offering to our church. To-day is Ash Wednesday, and as I write the nurses are working at the table, each looking so neat and nice in their uniforms, and the sun shining in upon us all like on a beautiful summer's day. One very bright feature of our Christmas was a loving letter from a nurse in England to her unknown sister nurses in British Columbia." And she ends her letter: "And now I must close with my Easter greeting to the nurses of England, and I must add how very pleased we are to receive a chance letter from any of them." How gladly we receive these far-away greetings our readers know well, and if our sister nurses thousands of miles away appreciate an English letter, tidings of them in their new lands and work find ever ready listeners and sympathy here.

### Notes and Queries.

**Queries.**

*M. E. W.*—Can any reader tell me the address of the Secretary of the Guild of St. Luke for young doctors?

**Answers.**

"*Sister*" thanks the numerous correspondents who offered a home for the epileptic patient, and hopes they will accept this acknowledgment, as it has been impossible for her to reply to all the letters received.

"*Sister,*" *Nurses' Home, Plaistow.*—You cannot do better than apply to the Matron, Manor House, Maghull, Liverpool. Here epileptic patients are received and most kindly treated. The house is large, and stands in its own grounds. It has before been named in these pages.—*Darenth.*

"*Pater.*"—The entrance and other fees at the various London hospitals vary in amount; and the same is true of the two Edinburgh medical schools, viz., the University and the extra-mural school. The proper course is to write to the Secretary of each London hospital; and of the University and extra-mural schools of medicine at Edinburgh, for a copy of the graduation regulations.

*S. B.*—Do you wish to know about the training at the various hospitals? Your question is not quite clear.

*M. A., Dundee.*—National Hospital, Queen Square; or Mrs. Creighton Hales, Maddox Street, W.

*Matron.*—The "Hospital Annual" would give you full information on your subject.

*E. J. P.*—You are under a misapprehension as to the necessity for doing what you mention; as you are a fully certificated nurse you are as eligible as anybody else.

*Nurses Endowed Bed.*—Two guineas collected by Nurse Goulter gratefully acknowledged.

### Among the Institutions.

**Newcastle Nurses' Home.**—The annual meeting of the Newcastle Nurses' Home shows things to be in a prosperous condition. This year sees them in a larger house, their old one being retained as a home hospital. There are now 59 nurses on the books and 10 probationers, and the committee have divided among them a sum of money as a bonus according to the time they have been there.

**Kingston District Nursing.**—Mr. Walter East took the chair at the Kingston Nursing Association's annual meeting. This Association is now affiliated with the Q.V.J. Institution, and shows a year of excellent progress, the epidemic having been a severe test to the nurses' capacities for getting through their work; 7,605 visits were paid during the year. The nurses are now Nurses Poynton and Lessels, and a trained probationer is soon to be added. We are glad to see that universal co-operation has been extended to the district nursing at Kingston, the churches and friendly societies having been most generous in their donations.

**Redruth Miners' Hospital.**—Perhaps there is no spot where a hospital can be more urgently needed than among a mining population where accident to life and limb may be expected daily, and nowhere is a nurse's care and work better appreciated. The Redruth Hospital was built by the late Lord Robartes on the recommendation of his agent, the late Mr. Alfred Jenkin, and the amount of good it has done is incalculable. Men brought almost to death's door by hard work, insufficient diet, and bad air, have by judicious treatment, thorough rest and generous diet, again and again been rapidly restored to health, and enabled to resume their duties as the bread winners of their several households, while in the accident wards the recoveries have been equally successful. Naturally, the hospital has a wonderful name in West Cornwall, it is a home replete with every comfort, and is thoroughly appreciated by the patients. There is no resident medical staff, the men choose their own doctor from among the mine doctors whom they pay monthly from their wages. The late Miss Angore and her sister, Miss Emily Angore, also dead, were matrons of this hospital from 1870 to the present, and Miss Edith Fry (Sister Edith) has taken the vacant post. The Ladies Committee of the Dartmouth Cottage Hospital, at their recent meeting, passed a unanimous vote of thanks to Miss Fry for her able management of the hospital, and expressed much regret at her resignation. The staff at Redruth remains the same, Nurse Kent being next in office to the matron. Over a hundred years ago, in his *Mineralogia Cornubiensis*, Dr. William Pryce, of Redruth, wrote the following paragraph; it will interest our readers to compare then and now, and probably the doctor's words laid an invisible stone of the Miners' Hospital many years before the visible building arose: "Now in the course of a year, the trepan or crooked knife will be wanted very often, beside the ordinary accidents of burns, wounds, contusions, or simple and compound fractures, where the knife is spared; the blasting of one or both eyes and the fingers of the left hand by gunpowder. When an accident happens in a mine, the poor sufferer languishes till the arrival of a surgeon, who is generally sent for in such haste and confusion he is not provided with everything proper to administer present relief. I have been called to a person supposed to have a compound fracture of the leg, by a fall twenty fathoms underground, and have brought suitable apparatus, when the case has proved to be a fractured skull and the leg only scratched. The patient is then conveyed six or seven miles to his own home, full of ill-clad children, and destitute of all conveniences and almost of all necessaries." We congratulate Miss Fry on her appointment to this interesting post; the enthusiastic Cornishmen seem to have given her a warm welcome.

### On Board the Antwerp Boat.

The steamer was to leave Antwerp at six p.m., and it was quite at the last moment, when the gangway was about to be pulled up, that George Carton, in his usual impetuous way, hurried on board, his hands full of sticks, bags, and wraps, and his pockets bulging to bursting with guide-books and the miscellaneous collection of an curiosity-loving Englishman during a three weeks foreign tour.

"Now then, sir. No time to lose."

"I haven't lost much, I flatter myself," said George, as he subsided breathlessly on to a seat. "I have seen a good deal more than most people in the time, and have managed to see three more churches in Antwerp instead of wasting an hour on board. Let's see; where's Badeker? I must look up that last place I saw. One does get horribly mixed up; and where are my photos of the Musée Plantin? I shouldn't like to lose those. Oh! I beg your pardon. I hope you are not hurt?" he exclaimed, as one of his weighty bags fell from the seat and nearly tripped up a young lady who was passing with an armful of shawls and cushions.

She steadied herself, but one of the cushions fell on the deck, and not having a hand to spare, she left it lying there until she could dispose of the other impedimenta. George could do nothing less than follow with the stray cushion to a sheltered corner of the deck, where a delicate-looking girl, seated in a camp-chair, was feebly and peevishly expostulating about the precautions the elder one was taking to shield her from cold.

"I shall be all right, Clare, I am not at all cold," she grumbled, "That shawl is too stuffy. I shall be stifled. I wish you would not fuss so. Why don't you go downstairs if you are so cold?"

"I am not cold, dear. I am only afraid you may feel the wind too much for you. We shall feel it more, too, when we get further down the river. Is your head better?"

"No, it's very bad. It makes me worse to talk."

"I will bring you some tea, dear. That may do it good."

But the invalid had closed her eyes and took no notice of the remark, or of the anxious attention with which she was watched. The elder sister stood doubtfully looking at the white face and worn, harassed look on the wasted features as if hesitating whether to leave her, when she suddenly realised that George was near still holding the cushion.

"Thank you, very much," she said simply. "I am sorry you had the trouble," and she turned once more to her sister's side.

George quickly divined her thoughts. "It is draughty round this corner, don't you think?" he said. "We can make that better perhaps in this way," and as he spoke he opened a large umbrella and fixed it behind the chair so as to screen the girl quite effectually.

"Oh, that is much better. I did not think of that. My sister is very sensitive to cold, but does not like to be taken care of. She thinks I am faddy. It makes it difficult for me to do what I should like."

They had moved over to the other side of the deck.

"Has your sister been ill long?" George asked. He felt a great interest in the sisters. The wistful expression of the elder one, so unconscious of herself, so completely wrapped up in her charge, seemed to lend another charm to her delicate and refined features.

"She would look awfully nice if she would smile," was George's mental comment.

But, perhaps at that moment the face of the younger sister attracted him still more, as the sunken features with the dull, weary expression, and the listless apathy of the figure aroused his professional interest.

"Not very long," Clare replied. "She was very ill six months ago, and since then she has been growing thinner and weaker. She eats so little, and takes no notice of anything. Oh, she is not the least the same." She spoke quickly and eagerly, as if it were a relief to talk to anyone of the anxiety preying on her mind. "The doctor says she must be roused by some means, and we have been abroad for a month, but she is no better. She will hardly speak to me sometimes. I wish I knew what to do! The doctor said some great shock might rouse her, but I think she would never get over it." She looked across at her sister, with tightly-clasped hands and tear-filled eyes.

"May I ask," George ventured to say, after a few minutes silence, "whether your sister has had a great trouble, or some mental strain. You must forgive my seeming inquisitive. I cannot call myself a doctor yet," he added, with a smile, which was the one attraction to a rather plain set of features. "But I am studying to become one, and I have seen some cases in which the mind has affected the health, and reduced the patient to much the same state as your sister is in."

"Ah! then you will understand," replied his companion more brightly. "It was a loss which made her so depressed at first. Our only brother had to go to Australia the beginning of the year, and she missed him dreadfully. They had always been together. He was never strong enough to go to school, and the doctor said the only chance of saving his life was the long sea voyage and a stay of some years in Australia. She had done so much for him all his life that the parting made her ill. She has never been the same since."

"Is there no one else at home?"

"No. I am away, and my father leads a very secluded life with his books, and is no companion to her. She so dreads going back to the loneliness at home that she begged to be allowed to go anywhere else. She says she would rather die than go back home again, and sometimes she talks so strangely I am afraid her mind is giving way."

Clare looked at her new acquaintance with an appealing glance. She had been obliged to think for herself and her sister so long that she eagerly caught at the opportunity of consulting someone else about her present difficulty, which seemed so insurmountable.

"You cannot go home with her at least for a short time, so that the first sense of loneliness may be less?"

"I wish I could, but it is quite impossible. I am training at St. Paul's Hospital, in London, and have already had more leave than I am entitled to. If I went home now I should lose my certificate, and that is so important for me, and I have worked for nearly three years."

George was silent. He felt that help was badly wanted, but he could do little or nothing to lighten the burden that pressed so heavily on the girl's young shoulders.

He could only turn her thoughts to the bright side, and remind her that she would soon be able to be at home again, and that the change would, no doubt, do her sister much good, although there seemed to be little benefit at present.

Then he began to talk of the places he had seen during his short holiday, and, taking out his sketch book, showed her some amusing scenes and characters he had met with on his travels.

She brightened up for a few minutes, and enjoyed his explanations, but suddenly grew pale and faint. "Would you get me a little water, please?"

He was gone before she had finished speaking.

"You will soon feel better," he said cheerfully. "The stewardess is bringing you some good soup. I feel sure you have eaten nothing to-day. Am I not right? You have forgotten yourself in looking after your sister. Take this, now you are faint with hunger and fatigue."

It seemed so natural to both of them that he should care for her in this way, and he soon saw himself repaid for his trouble by her reviving colour and improved appearance.

"There is a fresh breeze blowing," he said, "will you come to the end of the boat, it will revive you, and we shall have such a good view of the town. You ought to have a last look at the cathedral. It will be lovely with the evening light on it. There! now look back, this is one of the best views.

"Oh!" noticing her glancing anxiously towards the spot they had left. "I can see your sister quite well. She seems to be sleeping; that is the very best thing she can do."

In an instant she had sprung from her seat. A cry from a passenger, and her sister, starting round, saw a dark figure standing out in relief against the soft colours of the sunset sky, balancing for a moment on the edge of the vessel, then a splash in the dull waters below. She saw, as in a frightful dream, the figure by her side dart forward, cross the deck, and leap over the side.

She heard the second splash, which so quickly followed the first. She tried to move forward, staggered a few steps, and then remembered no more.

When Clare opened her eyes she found herself lying at full length on the deck seat. She looked up, wondering what had happened, and watched in a dreamy way the "Blue Peter" flapping overhead, until, as by a flash of lightning, she remembered. "Where was her sister; where was—— Oh! what had she been doing to lie there, when perhaps by that time——" Her brain swam as she struggled to her feet, and, with a deadly sinking at her heart, tried to collect herself to think clearly.

The kind voice of a fellow-passenger reassured her.

"Your sister is safe. They have taken her down to the ladies' cabin. You want to go to her. Let me help you; you feel faint still."

"No, I am quite strong. I must go," and, leaving her would-be helper, Clare hurried down the stairs, and in another moment was holding the trembling form of her sister in her arms.

The shock of the cold water, and the horrible feeling of sinking into the depths, had roused the mind of the sick girl from its apathy and melancholy. The love of life returned in full force, and she turned with a shudder from the thought of the death which she had so narrowly escaped. Passionately sobbing, she clung to her sister.

"Oh, Clare, can you forgive me? I did not mean to be so wicked. I thought there was nothing left to live for. I thought I did not want to live until I felt myself going down into that dreadful water. Oh, it was fearful;" and she trembled at the recollection. "If it had not been for that man I should have been drowned now. Oh, Clare, I will never be troublesome again. I will do what you like, and go where you like. You have been always good, and I know I have been very bad; but, Clare, my head *did* feel very bad sometimes, and I did not feel a bit like myself."

Clare caressed and soothed her like a tired child, and in spite of that awful moment, which she could not bear to think about, she felt happier and lighter-hearted than she had done for many months past. Her thoughts travelled from her sister to her new friend—no mere chance acquaintance now, but one to whom while she lived she would feel a deep and lasting sense of gratitude. "I can never thank him enough," she said to herself. "Never!"

But George felt that he was thanked enough when he saw the radiant look on Clare's face as she came up to him, and, putting her hand in his, tried, in a few broken words, to express the deep gratitude in her heart. He felt that he had brought back the happiness which had been so long absent from her life, for the tears in her eyes were not tears of sorrow.

He had to tell her over and over again how he had seized her sister's hair, and had kept her head above water until the boat had come to their help.

"And precious quick they were about it," he said, trying to turn her attention from his act of bravery to the promptitude of the crew, but she could not think of anyone else.

"Oh, if you had not been there," she repeated. "You don't know what you have done! You have saved her twice over—once from the sea, and again from that dreadful state she had fallen into. She will never be like that again. She is quite herself now. We owe it all to you," and so she repeated her thanks, and George honestly felt that he had been more than amply repaid, for he knew he had gained something which he would not lightly let go.

For the rest of the voyage he was the faithful aide-de-camp to the sisters. At the bustling station of Harwich he piloted them safely through the Custom House, that *bete noire* of ladies travelling by themselves. And not until they reached the dreary terminus of Liverpool Street Station were the final farewells said—or rather, we should say that there were no final farewells, for Clare's father insisted on making George's acquaintance, to thank him personally for having saved his child's life, and to express the wish that he might be able to make some return for the great service George had rendered him. And the old man kept his word when some years later George asked him for his elder daughter.

Dagmar.

## Figures and Tables

**Figure f1:**